# Drug information update. Atypical antipsychotics and neuroleptic malignant syndrome: nuances and pragmatics of the association

**DOI:** 10.1192/pb.bp.116.053736

**Published:** 2017-08

**Authors:** Siddharth Sarkar, Nitin Gupta

**Affiliations:** 1 All India Institute of Medical Sciences, New Delhi, India; 2 Government Medical College and Hospital, Chandigarh, India

## Abstract

Neuroleptic malignant syndrome (NMS) is a rare but potentially fatal adverse event associated with the use of antipsychotics. Although atypical antipsychotics were initially considered to carry no risk of NMS, reports have accumulated over time implicating them in NMS causation. Almost all atypical antipsychotics have been reported to be associated with NMS. The clinical profile of NMS caused by certain atypical antipsychotics such as clozapine has been reported to be considerably different from the NMS produced by typical antipsychotics, with diaphoresis encountered more commonly, and rigidity and tremor encountered less frequently. This article briefly discusses the evidence relating to the occurrence, presentation and management of NMS induced by atypical antipsychotics.

Neuroleptic malignant syndrome (NMS) refers to an idiosyncratic severe adverse reaction associated with the use of antipsychotics. It is a rare and unpredictable side-effect that has been associated with both first- and second-generation antipsychotics.^1,2^ It occurs in about 0.02–3% of individuals who are prescribed antipsychotics.^3^ NMS is generally characterised by rigidity fever, autonomic dysregulation, tremor, elevated creatine phosphokinase (CPK) levels and leucocytosis (Box 1).^4^ NMS is a potentially fatal adverse event. It can lead to permanent neurological impairment in survivors in the form of parkinsonian symptoms and cognitive deficits, which could be primarily ascribed to the raised core body temperature and ischaemia following rhabdomyolysis.^5–7^

Atypical (second-generation) antipsychotics were initially considered to have negligible risk of inducing NMS due to their distinctive pharmacodynamic characteristics.^8^ In fact, side-effect profile has been one of the important distinguishing features between typical (first-generation) and atypical antipsychotics. However, considerable research evidence has accumulated to suggest that atypical antipsychotics are also associated with NMS.^9,10^ Clozapine was one of the earliest atypical antipsychotics implicated in the causation of NMS.^11^ Subsequently, almost all of the atypical antipsychotics have been associated with the occurrence of NMS. The clinical features of NMS induced by atypical antipsychotics have been reported to be somewhat different from those induced by typical antipsychotics.^10^

Atypical antipsychotics are among the most commonly prescribed antipsychotics.^12,13^ They are utilised for the treatment of a range of psychiatric disorders including schizophrenia, mania, depression with psychotic symptoms and personality disorders, and for behavioural symptoms in dementia and intellectual disability.^14,15^ Hence, understanding the clinical presentation and occurrence of NMS associated with atypical antipsychotics is of clinical relevance. This article discusses the evidence relating to the occurrence of NMS with atypical antipsychotics and various aspects of management for this condition. The authors reckon that the definition of ‘atypical’ antipsychotics itself may not have clear margins,^16^ yet the term ‘atypical’ remains in clinical usage to refer to those medications that have comparably smaller chances of causing extrapyramidal symptoms. We do not aim to present a systematic review of the topic, but rather a pragmatic review of the literature on NMS with atypical antipsychotics.

## Reports of atypical antipsychotics causing NMS

### Risperidone

Risperidone has been associated with probably the largest number of cases of atypical antipsychotic-induced NMS.^9,17^ It has been noted more frequently in the younger age group who had been antipsychotic naive. A severe clinical picture of typical NMS has been encountered, marked by rigidity, extrapyramidal symptoms, fever and highly elevated CPK levels. Tachycardia was more common than diaphoresis and autonomic dysregulation occurred frequently.^9^

### Clozapine

Cases of clozapine-induced NMS, reported since the 1980s, typically occurred with rapid dose increases. Also, many patients who developed clozapine-induced NMS had a history of NMS with other antipsychotics. Tachycardia, tachypnoea, diaphoresis and autonomic lability were encountered frequently, possibly due to clozapine acting on adrenergic and muscarinic receptors.^18^ Rigidity and extrapyramidal symptoms were rare, possibly due to the lower affinity of clozapine to the D_2_ receptor. The increases in CPK were lower and delayed when compared with other antipsychotics. The occurrence of fever and autonomic instability in patients receiving clozapine in the absence of rigidity may necessitate ruling out the diagnosis of clozapine-related agranulocytosis before a diagnosis of NMS.^19^ The clinical severity of clozapine-induced NMS has been described to be lower than with other antipsychotics, and hence such cases have infrequently required intensive care unit admissions. The infrequent occurrence of rigidity and extrapyramidal symptoms in patients with clozapine-induced NMS require a high degree of suspicion for this diagnosis. However, certain researchers have suggested that clozapine-induced NMS should not be considered as a diagnosis in the absence of typical features of NMS.^20^

**Box 1** Symptoms and signs commonly encountered in neuroleptic malignant syndrome

- Fever
- Rigidity
- Elevated creatine phosphokinase (CPK) levels
- Tachycardia
- Tachypnoea
- Altered mental state
- Fluctuating blood pressure
- Diaphoresis
- Leukocytosis

### Olanzapine

Although olanzapine has been reported to present with the typical features of NMS, extrapyramidal symptoms and fever were absent in a small proportion of patients. Autonomic imbalances and diaphoresis are frequent, and are often the first signs to appear in patients with olanzapine-induced NMS. Nausea was infrequent, probably due to the antiemetic purported properties of olanzapine,^21^ but neurological impairments such as hemiplegia, ataxia and seizures have been reported.^9^ Several cases have been reported in patients receiving other medications apart from antipsychotics, for example mood stabilisers and antidepressants, and the clinical picture of NMS has been more severe in such patients.^9^

### Amisulpride

Several cases of amisulpride-induced NMS have been described in the literature,^22,23^ many reported in elderly males. The clinical profile primarily involves an altered mental state, frequent rigidity and high levels of CPK, whereas high fever, tremor and other autonomic symptoms have been reported less frequently. The lower propensity to cause autonomic symptoms is probably due to low affinity in amisulpride for muscarinic, adrenergic, serotonergic and histamine receptors than in other antipsychotics.^24^

### Quetiapine

Quetiapine-induced NMS has been primarily reported in the elderly, although it has also been described in children.^25^ Clinically, it presents with extrapyramidal symptoms and prominent autonomic symptoms such as tachycardia, blood pressure fluctuations, tachypnoea and diaphoresis. These prominent autonomic symptoms may be consequent to noradrenaline reuptake inhibition, histaminergic antagonism and serotonin toxicity associated with the use of quetiapine.^23^ The outcome of quetiapine-induced NMS has been relatively poor, probably due to the older age of patients in whom it has been reported.^9^

### Aripiprazole

Several case reports and case series have accumulated on the occurrence of NMS in patients receiving aripiprazole.^26,27^ Rigidity and altered mental state seem to be present frequently in such patients, while fever, diaphoresis and tachypnoea are less frequent. NMS has been reported to occur more commonly with fast upward titration of dosages of aripiprazole. The severity and duration of NMS seem lower than in other antipsychotic medications, probably due to the partial dopamine agonist activity of aripiprazole. Aripiprazole has also been implicated in combination antipsychotic regimens, when used alongside other atypical antipsychotics such as clozapine.^27^

### Ziprasidone

Few cases of ziprasidone-induced NMS have been described.^28,29^ The onset of NMS in these patients has been generally abrupt, with most displaying typical features such as alterations of mental state, fever, diaphoresis, tachycardia, blood pressure alterations, leukocytosis, tremor, and other extrapyramidal symptoms with high CPK. No fatality has been reported with ziprasidone to date, and recovery is usually achieved in about 10 days.

### Paliperidone

Paliperidone has a similar pharmacodynamic profile to risperidone, but it has a lower affinity for dopamine receptors and higher serotonin antagonist activity. Paliperidone-induced NMS has been described mainly in patients who have been previously treated with other atypical antipsychotics and have had a recent dose increase or cross-titrations.^30,31^ Paliperidone-induced NMS presents with a typical clinical profile with mental state alteration, rigidity, diaphoresis, hyperpyrexia, tremor and other extrapyramidal symptoms, and the outcome is favourable, with resolution achieved in all cases.

### Zotepine

Several cases of zotepine-induced NMS have been described in the literature.^22,32^ Rapid dose escalation was reported in one case, although NMS has also developed with the usual titration pattern. Zotepine-associated NMS presents with alterations of mental state, rigidity, diaphoresis, fever, tachycardia and leukocytosis, with less frequent occurrence of tremor, tachypnoea and alterations in blood pressure.

### Other atypical antipsychotics and summary

At present, there is a single case report of iloperidone being considered as a cause of NMS.^33^ The patient, who had schizophrenia, developed mutism, diaphoresis, diffuse lead pipe rigidity and tachycardia without fever or marked increase in CPK levels. The outcome was favourable, but the patient also required anticoagulation therapy for the management of comorbid pulmonary embolism.

Blonanserin was reported as a cause of NMS in a 30-year-old female with intellectual impairment.^34^ The patient presented with fever, tachycardia, rigidity, extrapyramidal symptoms and leukocytosis after the initiation of blonanserin. Symptomatic improvement was seen after discontinuation.

Although different atypical antipsychotics have different NMS clinical symptom profiles, rigidity, tremor and fever are encountered less frequently with atypical antipsychotics, whereas diaphoresis is quite common. Clozapine is particularly associated with atypical presentations of NMS with infrequent CPK level elevations. Risperidone, on the other hand, produces a clinical picture more similar to the NMS induced by typical antipsychotics. Some of the atypical antipsychotics have also been associated with serious features such as myoglobinuria and acute renal failure.^35,36^

## Risk factors for atypical antipsychotic-induced NMS

A few significant risk factors for atypical antipsychotic-induced NMS have been identified. They have been reported in one study as male gender, confusion, dehydration and delirium.^25^ Another study reported Black and minority ethnic background, antipsychotic polypharmacy, use of aripiprazole, and increasing dosing patterns.^37^ More recently, it has been suggested that rapid dose escalation of the antipsychotic may be a risk factor for NMS.^2^ The demographic profile of patients who developed NMS with atypical antipsychotics does not seem to differ substantially from that of patients with NMS induced by typical antipsychotics.

## Management

### Diagnostic uncertainty

The clinical picture and features of NMS with atypical antipsychotics seem to be different from those of typical antipsychotics. This had led to uncertainty over the diagnosis of NMS in patients on atypical antipsychotics who manifest only few of the NMS symptoms.^38^ Among the core symptoms of NMS, fever is often encountered less frequently in patients with atypical antipsychotic-induced NMS.^38^ The issue is further complicated by the various operational definitions of NMS.^38^ The DSM-IV-TR defines NMS as the presence of severe muscle rigidity and elevated temperature after antipsychotic initiation along with two or more of: diaphoresis, dysphagia, tremor, incontinence, changes in level of consciousness, mutism, tachycardia, elevated or labile blood pressure, leukocytosis, or laboratory evidence of muscle injury (elevated CPK level). Various other criteria for NMS have been postulated, each with varying emphasis on the individual symptoms and signs.^39^ Another set of criteria defines NMS in patients with either three major symptoms (hyperthermia, rigidity, elevated CPK level) or two major and four minor symptoms (diaphoresis, tachycardia, tachypnoea, abnormal blood pressure, leukocytosis, altered consciousness).^40^ Yet another diagnostic system defines NMS through the presence of extrapyramidal symptoms and fever (⩾37°C) alongside three minor symptoms within a 48-hour period.^41^ This may potentially mean that a case fulfilling the diagnosis of NMS according to one set of criteria may not do so with another set. The DSM-5 has taken a pragmatic approach of not explicitly stating the number of criteria required for the diagnosis of NMS.

It has been proposed that with the growing awareness of NMS, those in the early course of its development may benefit from early identification and immediate treatment. This may lead to an abortive course of NMS development, with an incomplete picture and only few of the criteria being met. Hence, some authors have proposed a dimensional concept of NMS, which takes into consideration the minor and subthreshold forms of NMS.^38,42^ This is likely to further our knowledge about NMS pathophysiology, clinical profile subtypes and appropriate management strategies.

Furthermore, various other medical and neurological conditions may present with a clinical picture similar to NMS (briefly mentioned in Box 2). Patient condition may require expedient decisions so that a rational line of management can be instituted. Hence, the clinician may need to take a brief and focused history for being reasonably sure about the diagnosis. Neuroimaging and electroencephalogram may be helpful for ruling out neurological pathologies mimicking NMS. For example, in patients with psychosis, catatonia may be considered as a differential diagnosis, especially when the patient is mute and exhibits staring. It may not be possible to exhaustively rule out all differential diagnoses, and at times management may need to be started on an empirical basis.

## Treatment strategies for NMS

The management of NMS caused by atypical antipsychotics would not be substantially different from the management of NMS induced by typical antipsychotics (Box 3). NMS is a medical emergency and requires immediate attention for clinical management. Clinical diagnosis should be supplemented by laboratory tests, particularly CPK levels and total leukocyte counts. Once the diagnosis is suspected, the offending antipsychotic agent must be immediately stopped. Regular monitoring of the vitals should be carried out. The patient should be moved to the intensive care unit based on the severity of their medical condition. Intensive care would typically focus on monitoring of cardiorespiratory and renal status. Serial monitoring of serum electrolytes should be performed and corrected as required. In extreme hyperthermia, physical cooling measures may be instituted.

Several pharmacological options are available for the treatment of NMS.^43,44^ Dopaminergic agents such as amantadine and bromocriptine have been demonstrated to decrease the duration of and mortality associated with NMS. Amantadine 200 mg to 400 mg per day in divided doses is administered either through a nasogastric tube or orally. Bromocriptine is started at the dose of 2.5 mg three times a day and can be titrated upwards to 45 mg per day. Benzodiazepines, particularly lorazepam, can be given when underlying catatonia is suspected and where agitation is encountered in the patient. Lorazepam challenge can be done with 1 to 2 mg lorazepam administered parenterally, and may be continued in cases which show some response. Dantrolene is a muscle relaxant that can be applied in cases presenting with severe rigidity and hyperthermia. It is initiated at doses of 1–2.5 mg/kg body weight and can be repeated 6 hourly if improvement is seen. It can be administered orally after improvement with the parenteral preparation.

**Box 2** Differential diagnosis of neuroleptic malignant syndrome

- Amphetamine toxicity
- Anticholinergic delirium
- Benign extrapyramidal side-effects
- Brain abscess
- Catatonia
- Heat stroke
- Malignant catatonia
- Malignant hyperthermia
- Meningitis or encephalitis
- Mid-brain structural lesions
- Non-convulsive status epilepticus
- Sepsis
- Serotonin syndrome
- Thyrotoxicosis

The altered mental state encountered during NMS also needs attention. If sedation is required, benzodiazepines may be a preferred choice. The medical management of the patient takes precedence over the underlying psychiatric disorder. As the patient's condition improves, discussion about further treatment options may be initiated.

## Re-challenge with antipsychotics after NMS

One of the important considerations for a clinician is to whether to start another antipsychotic after a patient develops NMS. If the antipsychotic had been started for the control of psychotic symptoms, then the risk of psychosis without the cover of antipsychotics is high. The clinician may have to weigh the pros and cons of re-starting antipsychotic medication: the advantage of making the patient more manageable against the risk of inducing NMS.

**Box 3** Treatment of neuroleptic malignant syndrome

- Consider shifting to intensive care unit
- Regular monitoring of vitals
- Monitoring of electrolytes and correction if required
- Management of medical comorbidity
- Physical cooling measures if required
- Dopaminergic medications: amantadine and bromocriptine
- Muscle relaxant: dantrolene
- Benzodiazepines: for management of agitation, when clinical suspicion of catatonia is present

Several reports of post-NMS antipsychotic re-challenge have been published.^45–48^ Indications for a re-challenge need to be clearly documented and other options of management (including electroconvulsive therapy) should be explored. Taking informed consent from the patient and/or family members/carers becomes necessary in such circumstances. Re-challenge should be done with an atypical antipsychotic with low propensity to cause NMS and dose titration should be gradual. Careful monitoring should be instituted, watching the evolution of symptoms of NMS. The re-challenge strategies thus adopted are in no way different from those post-NMS due to typical antipsychotics.

## Conclusions

As psychiatrists, we are likely to encounter NMS induced by atypical antipsychotics during clinical practice. Although it is an uncommon adverse event of antipsychotic use, the potential fatality requires the clinician to take cognisance of this, and institute treatment immediately. The presentation of NMS induced by atypical antipsychotics, especially clozapine, may be quite different from NMS induced by typical antipsychotics – rigidity and tremor are encountered less frequently, while diaphoresis is probably encountered more frequently. Hence, a high degree of clinical suspicion may be required. Overall, the management of NMS induced by atypical antipsychotics is not broadly different to the management of that induced by typical antipsychotics. Additionally, an episode of resolved NMS does not preclude the subsequent initiation of antipsychotics, although due caution needs to be exercised while re-challenging antipsychotics in patients with a history of NMS induced by atypical antipsychotics.
